# Percutaneous drainage for patients with cervical necrotizing fasciitis with novel CT classification based on extension of fluid collection along the deep cervical space

**DOI:** 10.1186/cc14093

**Published:** 2015-03-16

**Authors:** T Kiguchi, S Fujimi

**Affiliations:** 1 Osaka General Medical Center, Osaka, Japan

## Introduction

Cervical necrotizing fasciitis (CNF) is a rapidly evolving and life-threatening condition. Therefore, it is important for physicians to evaluate the severity of illness and to predict clinical outcome exactly in the early phase. We focused on extension of acute fluid collection along the deep cervical space by CT findings. The purpose of this study was to produce the CT grade and to analyze whether our CT grade is related to the clinical features and the responses to treatment of CNF.

## Methods

Between June 2004 and December 2012, 42 patients diagnosed and treated for CNF in two institutions were included in this study. Cervical spaces were subdivided into three components according to the concept of interfascial planes. The extension of acute fluid collection in cervical spaces was classified into three grades: Grade I, fluid collection confined to one component; Grade II, fluid collection spreading into two or three components; and Grade III, fluid collection spreading into four components or mediastinum. We analyzed association with CT grades and severity of illness (SOFA score, APACHE II score, CRP). All patients underwent percutaneous catheter drainage either ultrasonography guided or CT guided. We compared treatment outcome of CNF with CT grades.

## Results

According to elevation of CT grades, severity of illness was significantly associated with high score (APACHE II: 10.5 to 4.0, 12.8 to 4.2, 16 to 4.2, SOFA: 2.6 to 1.5, 2.9 to 1.9, 6.8 to 3.7, CRP: 17.8 to 10.6, 22.4 to 10.1, 33.3 to 11.9) and also duration of mechanical ventilation and length of hospital stay were longer (duration of mechanical ventilation: 10.9 to 6.6, 11.5 to 6.7, 15.8 to 7.2, length of hospital stay: 23.4 to 10.6, 27.9 to 21.4, 48.7 to 36.2).

## Conclusion

Novel classification of CNF based on CT findings showing the extension of fluid collection is a useful indicator of the disease severity and predicting clinical outcome. These findings may influence the strategy for the success of percutaneous catheter drainage.

